# Intraoperative Clinical Examination for Assessing Pelvic and Para-Aortic Lymph Node Involvement in Advanced Epithelial Ovarian Cancer: A Systematic Review and Meta-Analysis

**DOI:** 10.3390/jcm9092793

**Published:** 2020-08-29

**Authors:** Camille Mimoun, Jean Louis Benifla, Arnaud Fauconnier, Cyrille Huchon

**Affiliations:** 1 Department of Gynecology and Obstetrics, Lariboisiere Hospital, 75010 Paris, France; mimouncamille@gmail.com (C.M.); jlb@gmail.com (J.L.B.); 2 Research Unit EA 7285 “Risk and Safety in Clinical Medicine for Women and Perinatal Health”, University of Versailles Saint-Quentin (UVSQ), 78300 Poissy, France; arnaud.fauconnier@ght-yvelinesnord.fr; 3 Department of Gynecology, Poissy-St. Germain Hospital, 78300 Poissy, France

**Keywords:** intraoperative clinical examination, lymph node metastases, advanced epithelial ovarian cancer, diagnostic accuracy, systematic review, meta-analysis, meta-regression

## Abstract

After the publication of the Lymphadenectomy in Ovarian Neoplasms (LION) trial results, lymphadenectomy in advanced epithelial ovarian cancer with primary complete cytoreductive surgery is considered indicated only for women with suspicious lymph nodes. The aim of this meta-analysis was to evaluate the diagnostic accuracy of intraoperative clinical examination for detecting lymph node metastases in patients with advanced epithelial ovarian cancer during primary complete cytoreductive surgery. MEDLINE, EMBASE, Web of Science and the Cochrane Library were searched for January 1990 to May 2019 for studies evaluating the diagnostic accuracy of intraoperative clinical examination for detecting lymph node metastases in patients with advanced epithelial ovarian cancer during primary complete cytoreductive surgery, with histology as the gold standard. Methodological quality was assessed by using the QUADAS-2 tool. Pooled diagnostic accuracy was calculated, and hierarchical summary receiver operating curve was constructed. The potential sources of heterogeneity were analyzed by meta-regression analysis. Deek’s funnel plot test for publication bias and Fagan’s nomogram for clinical utility were also used. This meta-analysis included five studies involving 723 women. The pooled sensitivity of intraoperative clinical examination for detecting lymph node metastases was 0.79, 95% CI (0.67–0.87), and its specificity 0.85, 95% CI (0.67–0.94); the area under the hierarchical summary receiver operating curve was 0.86, 95% CI (0.83–0.89). In the meta-regression analysis, patient sample size, mean age, and type of cancer included were significant covariates explaining the potential sources of heterogeneity. Deek’s funnel plot test showed no evidence of publication bias (*p* = 0.25). Fagan’s nomogram indicated that intraoperative clinical examination increased the post-test probability of lymph node metastases to 79% when it was positive and reduced it to 16% when negative. This meta-analysis shows that the diagnostic accuracy of intraoperative clinical examination during primary complete cytoreductive surgery for detecting lymph node metastases in advanced epithelial ovarian cancer is good.

## 1. Introduction

Ovarian cancer is the fifth leading cause of cancer death in the United States, with an estimated 22,530 new cases diagnosed and 13,980 deaths in 2019 [1]. The mainstay of treatment of advanced epithelial ovarian cancer consists of primary complete cytoreductive surgery followed by chemotherapy with carboplatin and paclitaxel [2].

Although the completeness of cytoreductive surgery has a demonstrated and substantial survival benefit [3], the utility of systematic pelvic and para-aortic lymphadenectomy in advanced epithelial ovarian cancer has been recently questioned, despite evidence that lymph node invasion is a major prognostic factor [4,5,6,7]. Indeed, older retrospective studies suggested this systematic lymphadenectomy had potential survival benefits [7,8,9,10,11,12], but two recent prospective randomized trials failed to prove it [4,13], in particular the Lymphadenectomy in Ovarian Neoplasms (LION) trial published in 2019. It considered only advanced epithelial ovarian cancer with primary complete cytoreductive surgery and clinically negative lymph nodes and reported that neither overall nor progression-free survival was longer in the group associated with systematic lymphadenectomy compared with the group with no lymphadenectomy [13].

The 2019 French guidelines issued after the LION trial results were published accordingly restricted the indication for pelvic and para-aortic lymphadenectomy in advanced epithelial ovarian cancer with primary complete cytoreductive surgery to women with suspicious lymph nodes, in particular, on intraoperative clinical examination [14]. If the surgical team considers them metastatic, lymphadenectomy will take place—if not, it will not. Thus, surgical treatment management depends mainly on intraoperative clinical examination. This systematic review and meta-analysis will evaluate the diagnostic accuracy of intraoperative clinical examination for detecting pelvic and para-aortic lymph node metastases in patients with advanced epithelial ovarian cancer during primary complete cytoreductive surgery. No survival analysis was performed in this study, but only a diagnostic test accuracy review and meta-analysis.

## 2. Materials and Methods

### 2.1. Protocol and Registration

**Figure A1:**
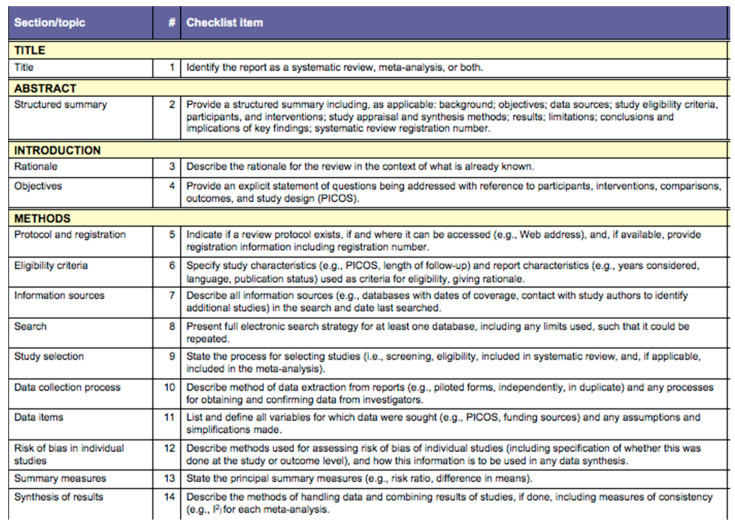
PRISMA checklist.

This systematic review and meta-analysis were conducted according to the PRISMA guidelines (Preferred Reporting Items for Systematic Reviews and Meta-Analyses) [15]; a detailed PRISMA checklist can be found in Figure A1 (Appendix A). The protocol was registered in the PROSPERO international prospective register of systematic reviews and information about it is available at http://www.crd.york.ac.uk/prospero/, no. CRD42020136395. No ethical approval or written, informed consent was required.

### 2.2. Data Sources and Search Strategy

A comprehensive search of the MEDLINE (PubMed), EMBASE, Web of Science and Cochrane Library databases looked for relevant published articles about the diagnostic accuracy of intraoperative clinical examination for detecting pelvic and para-aortic lymph node metastases in patients with advanced epithelial ovarian cancer during primary complete cytoreductive surgery. We restricted our search to the period from January 1990 to May 2019 and studies in English and French. Reference lists and citation sections of the retrieved articles were also screened for additional eligible studies.

The search in those databases used combinations of the following keywords: ([“ovarian cancer”] OR [“ovarian neoplasm”] OR [“ovarian carcinoma”] OR [“ovarian tumor”] OR [“ovarian tumour”]) AND ([“lymph node”] OR [“nodal”] OR [“metastases”] OR [“metastasis”]) AND ([“accuracy”] OR [“diagnostic value”] OR [“diagnostic performance”] OR [“diagnosis”] OR [“sensibility and specificity”]).

### 2.3. Selection Criteria

The following inclusion criteria were used to select articles for this study: (1) Original studies evaluating the diagnostic accuracy of intraoperative clinical examination for detecting pelvic and para-aortic lymph node metastases in patients with advanced epithelial ovarian cancer (FIGO 2018 2B-4A); (2) primary complete cytoreductive surgery, including pelvic and para-aortic lymphadenectomy, with histopathological examination of the nodes serving as the gold standard; and (3) studies reporting data necessary to build 2 × 2 contingency tables with the absolute numbers of true-positive, false-positive, true-negative, and false-negative cases. Studies were excluded if they focused on women with either recurrent ovarian cancer or surgery after chemotherapy.

### 2.4. Study Selection

Two reviewers (Camille Mimoun and Cyrille Huchon) independently selected the studies. After removal of duplicates, the reviewers began by reading titles and abstracts. If the study appeared relevant, the full text was then assessed for eligibility based on our inclusion and exclusion criteria. Finally, a selection of articles for the meta-analysis was made. Disagreements were resolved by consensus.

### 2.5. Data Extraction and Quality Assessment

One reviewer (Camille Mimoun), using a standardized data extraction form, recorded data from each selected study, and a second reviewer (Cyrille Huchon) checked them. The following data were extracted: Author, year and country of publication, study characteristics (design, number of centers, inclusion interval dates), inclusion and exclusion criteria (type of cancer included, histology, FIGO stage, initial/complete surgery), gold standard and results, pelvic and para-aortic lymphadenectomy protocol, intraoperative clinical examination protocol and results, number of patients and/or number of nodes, patient’s mean age, and the data necessary to build 2 × 2 contingency tables (number of true-positive, false-positive, true-negative, and false-negative cases).

To verify the quality of eligible studies, two reviewers (Camille Mimoun and Cyrille Huchon) independently analyzed the risk of bias and applicability concerns for four domains (patient selection, index test, reference standard, flow and timing) with the QUADAS-2 tool (Quality Assessment of Diagnostic Accuracy Studies) [16]. QUADAS-2 was performed with Review Manager 5.2.

### 2.6. Statistical Analysis

Bivariate random-effects models [17] were used to generate pooled summary estimates of sensitivity, specificity, positive likelihood ratios (LR+), negative likelihood ratios (LR−) and diagnostic odds ratios (DOR) with their 95% confidence intervals (Cis) of intraoperative clinical examination for detecting pelvic and para-aortic lymph node metastases from the number of true-positive, false-positive, true-negative, and false-negative cases extracted from each individual study. A hierarchical summary receiver operator characteristic curve was constructed with the pooled sensitivity and specificity values to obtain the area under the curve, which reflects the overall accuracy of intraoperative clinical examination for detecting pelvic and para-aortic lymph node metastases [18]. The heterogeneity of the pooled studies was assessed with Cochran’s Q test and the I^2^ index (I^2^ > 50% was considered substantial heterogeneity) [19]. Meta-regression analysis was performed to investigate the potential sources of heterogeneity in the studies (study design, patient sample size, patient’s mean age and type of cancer included). We used Deek’s asymmetry test with a funnel plot to detect publication bias [20] and Fagan’s nomogram to evaluate the clinical utility of intraoperative clinical examination [21].

The “midas module” [22] for meta-analysis of diagnostic accuracy studies was used in STATA version 13.1 (College Station, TX, USA). *P*-values less than 0.05 were considered statistically significant.

## 3. Results

### 3.1. Study Selection

**Figure 1:**
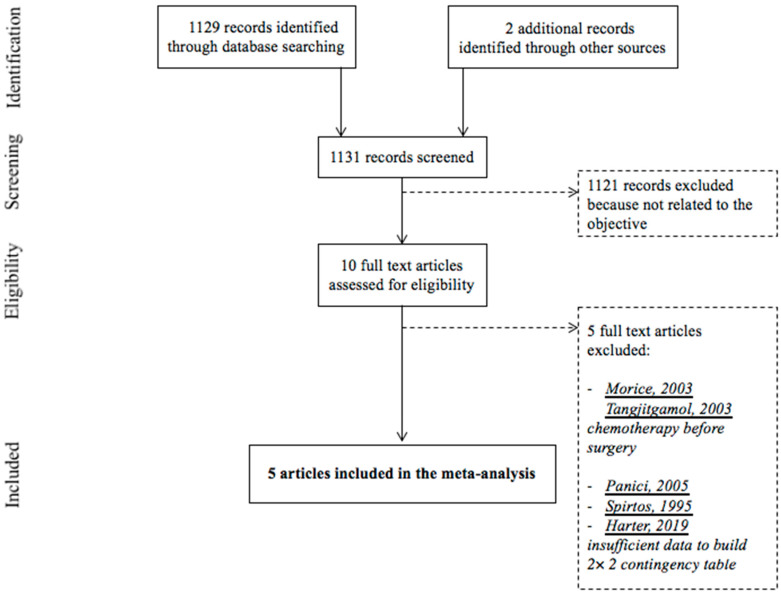
Flow chart of the study selection process.

Figure 1 presents the flow chart of the study selection process. The initial search results produced 1131 articles (1129 articles identified through database searching and two articles identified through other sources). After screening based on title and abstract review, 10 articles were read in full to assess their eligibility [4,13,23,24,25,26,27,28,29,30]. Finally, the meta-analysis included five studies [23,24,25,26,27].

### 3.2. Study Description

Table 1 summarizes the characteristics of the studies and participants included in the meta-analysis. The pelvic and para-aortic lymphadenectomy and intraoperative clinical examination protocols are summarized in Table A1. These studies, all published in English between 2000 and 2007, including a total of 723 women. They all evaluated the diagnostic accuracy of intraoperative clinical examination for detecting pelvic and para-aortic lymph node metastases, and they all used histology as the gold standard. All five studies were analyzed on a per-patient basis.

### 3.3. Quality Assessment

**Figure 2:**
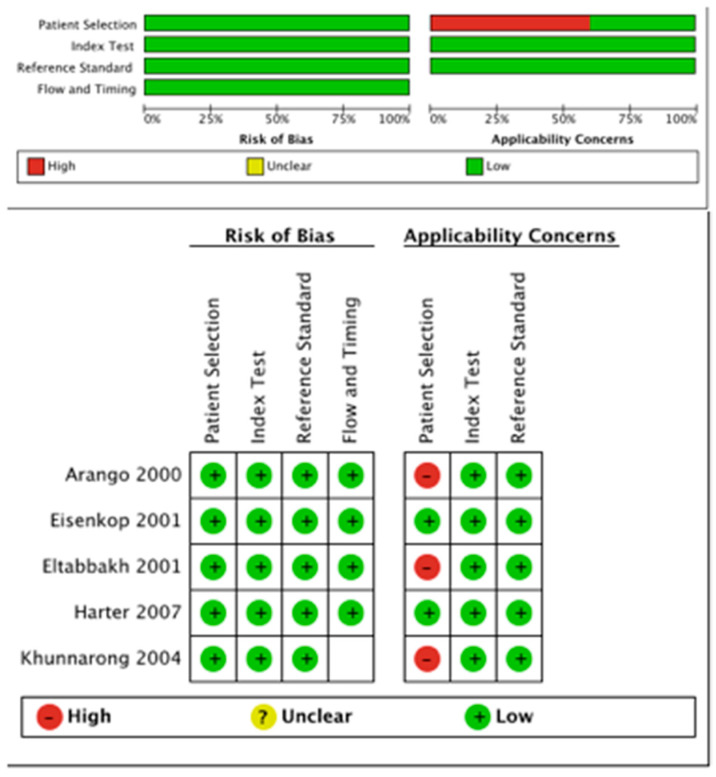
QUADAS-2 risk of bias and applicability concerns.

The methodological quality of studies included in the meta-analysis is presented in Figure 2. The quality of these studies was high for all four domains. The principal risk of bias related to applicability concerns for the patient selection domain in three studies [23,25,26]. Specifically, these studies included not only ovarian but also other gynecologic cancers. Furthermore, the authors did not describe histology, FIGO stage, and if the surgery was primary and/or complete for the ovarian cancers included in these studies.

### 3.4. Statistical Analysis

#### 3.4.1. Diagnostic Accuracy of Intraoperative Clinical Examination

**Figure 3:**
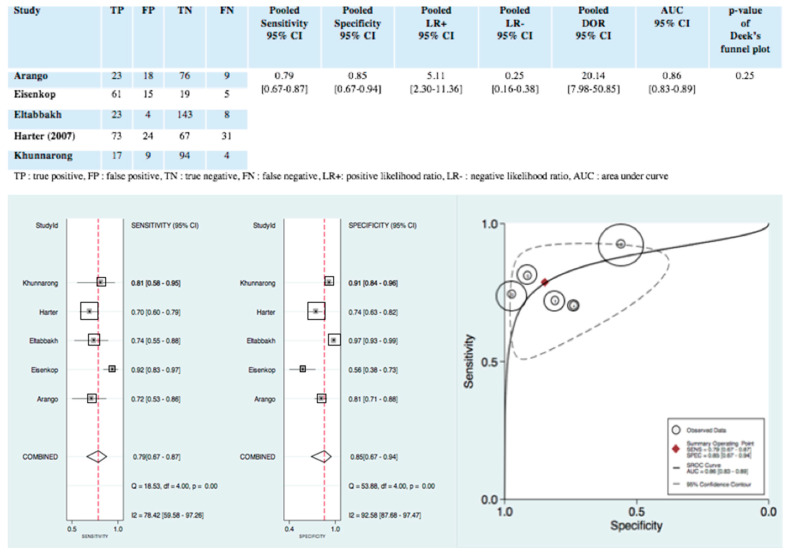
Diagnostic accuracy, forest plot of pooled sensitivity and specificity AND hierarchical summary receiver operating curve of intraoperative clinical examination for detecting pelvic and para-aortic lymph node metastases. Forest Plot: Each square represents one included study, and the size of each square reflects the sample size of each included study. Error bars represent 95% Cis. The “combined” values represent the pooled sensitivity and pooled specificity. I^2^ > 50% indicated substantial heterogeneity in the diagnostic parameters across studies. HSROC: Each circle represents one included study, and the size of each circle reflects the sample size of each included study. Values in brackets are 95% Cis. AUC is the area under the curve. 1: Khunnarong; 2: Harter; 3: Eltabbakh; 4: Eisenkop; 5: Arango.

Figure 3 shows the pooled results of the diagnostic accuracy of intraoperative clinical examination for detecting pelvic and para-aortic lymph node metastases. Figure 3 also presents the forest plots of pooled sensitivity and specificity, respectively, 0.79, 95% CI (0.67–0.87) and 0.85, 95% CI (0.67–0.94). Pooled positive likelihood ratio and pooled negative likelihood ratio were, respectively, 5.11 95% CI (2.30–11.36) and 0.25 95% CI (0.16–0.38). The pooled diagnostic odd ratio was 20.14 95% CI (7.95–50.85). The hierarchical summary receiver operating curve in Figure 3 shows an area under the curve of 0.86, 95% CI (0.83–0.89). Heterogeneity was substantial for sensitivity and specificity, respectively, Q = 18.53; *p* = 0.00; I^2^ = 78.42, 95% CI (59.58–97.26) and Q = 53.88; *p* = 0.00; I^2^ = 92.58, 95% CI (87.68–97.47) (Figure 3).

#### 3.4.2. Exploration of Heterogeneity

**Figure 4:**
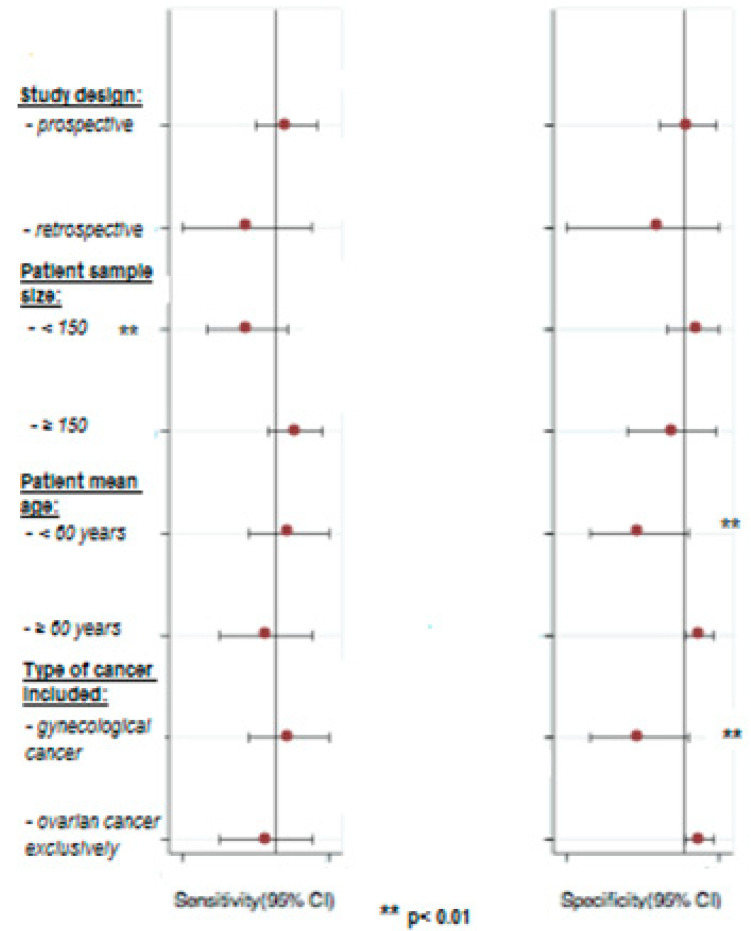
Univariate meta-regression analysis for identifying heterogeneity in studies included in the meta-analysis. The meta-regression shows that patient sample size could be the source of heterogeneity for sensitivity (meta-regression *p* < 0.01) and that patient’s mean age and type of cancer included could be sources of heterogeneity for specificity (meta-regression *p* < 0.01).

Figure 4 shows the results of the univariate meta-regression analysis. Study design (prospective vs. retrospective), patient sample size (<150 vs. ≥150), patient’s mean age (<60 vs. ≥60) and type of cancer included (gynecologic cancer vs. ovarian cancer exclusively) were used as covariates. Patient sample size could be the source of heterogeneity for sensitivity (meta-regression *p* < 0.01). The patient’s mean age and type of cancer included could be sources of heterogeneity for specificity (meta-regression *p* < 0.01). The specificity of intraoperative clinical examination for detecting pelvic and para-aortic lymph node metastases in women with ovarian cancer, exclusively, was 0.92, 95% CI (0.85–0.98).

#### 3.4.3. Publication Bias

**Figure A2:**
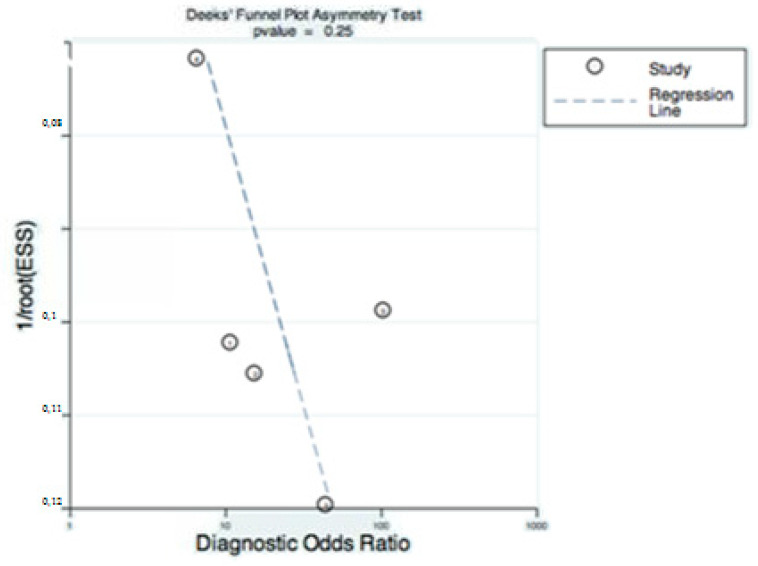
Deek’s funnel plot asymmetry test of intraoperative clinical examination for detecting pelvic and para-aortic lymph node metastases. 1: Khunnarong; 2: Harter; 3: Eltabbakh; 4: Eisenkop; 5: Arango; ESS: effectif sample size. Symmetrically distributed studies with the regression line and *p*-value of 0.25 indicate no publication bias.

Deek’s funnel plot asymmetry test showed no statistical evidence of publication bias (*p* = 0.25) (Figure A2).

#### 3.4.4. Clinical Utility

**Figure A3:**
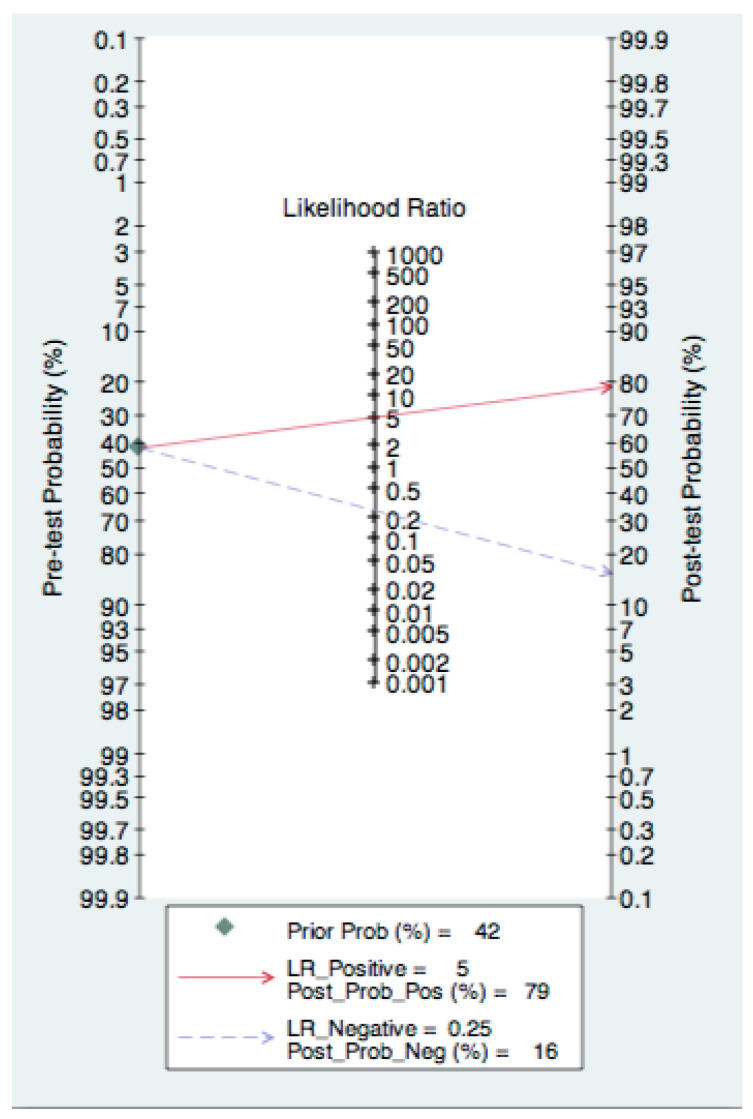
Fagan’s nomogram for likelihood ratios and the probability of intraoperative clinical examination for detecting pelvic and para-aortic lymph node metastases. The Fagan’s nomogram shows that, with a pre-test probability of lymph node metastases as in our sample of 42%, a positive intraoperative clinical examination gives a post-test probability of 79%, while a negative intraoperative clinical examination gives a post-test probability of 16%.

Figure A3 shows Fagan’s nomogram for likelihood ratios. The lymph node metastases pre-test probability was 42%. The nomogram indicated that intraoperative clinical examination increased the lymph node metastases post-test probability to 79% when it was positive and reduced the lymph node metastases post-test probability to 16% when it was negative.

## 4. Discussion

Intraoperative clinical examination is one of the diagnostic tools used by gynecologic oncologists for patients with advanced epithelial ovarian cancer during primary complete cytoreductive surgery for detecting pelvic and para-aortic lymph node metastases, and thus, to decide if they should perform lymphadenectomy. We conducted the first systematic review and meta-analysis to evaluate the diagnostic accuracy of intraoperative clinical examination for detecting pelvic and para-aortic lymph node metastases. No survival analysis was performed in this study, but only a diagnostic test accuracy review and meta-analysis.

This meta-analysis includes five studies and 723 women. It has several strengths. We followed a standardized protocol with a comprehensive search strategy, study selection, and data extraction. Four of the five studies included in the meta-analysis were prospective. The quality of the five included studies showed a low-risk of bias for the four domains (patient selection, index test, reference standard, flow and timing); in particular, all the lymph node metastases were confirmed by histology and enabled a misclassification bias to be ruled out. Bivariate random-effects models and hierarchical summary receiver operating curve were used; the patient samples were pooled so that the findings of this meta-analysis are more robust than any of the individual studies.

However, this meta-analysis has one major limitation. Only five studies were included because the literature is sparse, and our inclusion criteria were very strict. Moreover, three of the five articles included not only ovarian cancer, but also endometrial, cervical, vaginal and vulvar cancers [23,25,26]. This issue explains the high risk of bias concerning the applicability concerns for patient selection in those three studies.

Pooled sensitivity was 0.85, 95% CI (0.67–0.94) and pooled LR− was 0.25, 95% CI (0.16–0.38). Our meta-analysis, therefore, confirmed that women with the negative intraoperative clinical examination are a group at low risk of lymph node metastases (LR− ≤ 0.25) [31]. Pooled specificity was 0.79, 95% CI (0.67–0.87) and pooled LR+ was 5.11, 95% CI (2.30–11.36). The selection bias, induced by the inclusion in our meta-analysis of three studies (which included not only ovarian cancers, but also other gynecological cancers), was analyzed by the meta-regression analysis. Indeed, the type of included cancers could be the source of heterogeneity for specificity. However, the result for our meta-analysis is very interesting, because in our population of patient with advanced epithelial ovarian cancer the pooled specificity was significantly higher than the global pooled specificity (0.92 95% CI (0.85–0.98) vs. 0.79, 95% CI (0.67–0.87), *p* < 0.01). Our meta-analysis, therefore, confirmed that patients with the positive intraoperative clinical examination are a group at high risk of lymph node metastases (LR+ ≥ 4.0) [31]. The area under the curve of the hierarchical summary receiver operating curve was 0.86, 95% CI (0.83–0.89), which indicates that the accuracy of intraoperative clinical examination for detecting pelvic and para-aortic lymph node metastases is high.

This meta-analysis confirms the important message of the LION trial for the routine clinical practice of gynecologic oncologists:

- Positive intraoperative clinical examination triages patients into a group at high risk of lymph node metastases, with a clear indication for pelvic and para-aortic lymphadenectomy, as the literature, and particularly the LION trial, recommends;
- Negative intraoperative clinical examination triages patients into a group at low risk of lymph node metastases. However, it does not appear sufficient to conclusively rule them out, in view of the high number of false-negative of intraoperative clinical examination in the five studies included in our meta-analysis Spirtos et al. [28] and Harter et al. [13], which were excluded from the meta-analysis because their reports lacked the data to build a complete contingency 2 × 2 table, also found a high number of false-negative of intraoperative clinical examination—13/56 patients (23.2%) and 180/323 (55.7%) patients, respectively. Nonetheless, it must be noted that in the LION trial despite the 55.7% of a false-negative, no survival difference between the “lymphadenectomy” group and the “no lymphadenectomy” group was shown.

In conclusion, this systematic review and meta-analysis demonstrates the high diagnostic accuracy of intraoperative clinical examination for detecting pelvic and para-aortic lymph node metastases in advanced epithelial ovarian cancer during complete cytoreductive surgery. As the LION trial demonstrated, our meta-analysis confirms that intraoperative clinical examination is a safe diagnostic tool to determine if patients with advanced epithelial ovarian cancer should have lymphadenectomy during complete cytoreductive surgery.

## Figures and Tables

**Table 1:** Characteristics of studies and participants.

Author Year Country	Study Design	Number of Centers	Inclusion Intervals	Type of Cancer	Histology	FIGO Stage	Initial/Complete Surgery	Gold Standard	Number of Patients/Nodes	Patients’ Mean Age (Years)
**Arango** 2000 US	Prospective	1	August 1995–June 1997	-Ovarian (21%) -Cervical (43%) -Uterine (31%) -Vaginal (2%)	-	-	-	Histology	126/2138	55 (range, 18–83)
**Eisenkop** 2001 US	Prospective	1	1997–2000	Ovarian	Epithelial	IIIC and IV	Primary complete cytoreductive surgery	Histology	100/-	61.4 (range, 24.2–88.3)
**Eltabbakh** 2001 US	Prospective	1	February 1998–September 1999	-Ovarian (30.9%) -Endometrial (41%) -Cervical or vaginal (19.1%) -Vulvar (9%)	-	-	-	Histology	178/2158	56.6 (range, 18–90)
**Harter** 2007 Germany	Retrospective	1	2000–2005	Ovarian	Epithelial	-Early ovarian cancer (36%) -Advanced ovarian cancer (IIIb to IV) (64%)	Primary complete cytoreductive surgery	Histology	195/-	60 (range, 22–80)
**Khunnarong** 2004 Thailand	Prospective	1	May 2003–April 2004	-Ovarian (17%) -Cervical (48%) -Endometrial (33%) -Vulvar (2%)	-	-	-	Histology	124/1609	51 ± 11.3

- Data not available.

## Appendix B

**Table A1:** Pelvic and para-aortic lymphadenectomy and intraoperative clinical examination protocols.

Study	Surgeons Qualifications	Lymphadenectomy Protocol			Intraoperative Clinical Examination Protocol
		Type	Indication	Limits/Regions	
Arango	3 obstetrician-gynecologist with subspecialty certification in gynecologic oncology from the American Board of Obstetrics and Gynecology, who had practiced gynecologic oncology for at least five years	-Pelvic or para-aortic lymph node samplings -Pelvic and para-aortic lymphadenectomies	-FIGO stage 1a and 1b cervical cancer had lymphadenectomies -Decision on whether to do lymphadenectomies or lymph node samplings in all other cancers were left to individual surgeons	-Pelvic lymphadenectomy: the deep circumflex iliac veins distally, the obturator nerves inferiorly, the internal iliac arteries medially and the aortic bifurcation proximally -Para-aortic lymphadenectomy: bifurcation of the aorta distally and the inferior mesenteric artery proximally, left and right sides were included	-Data of lymph node status were collected on gynecologic oncologist’s opinions by palpation of open retroperitoneal spaces -Nodes believed to be positive were sent separately
Eisenkop	-	Systematic pelvic and aortic lymphadenectomy	Advanced ovarian cancer + primary cytoreductive surgery	-Pelvic lymphadenectomy: removal of all identifiable nodal tissue bilaterally associated with the common iliac, external iliac, hypogastric vessels and the obturator fossa -Aortic lymphadenectomy: removal of all identifiable nodal tissue from the aortal-caval region, the lateral and anterior surface of the aorta and vena cava to the approximate levels of the renal vessels	Nodes were classified to be positive by palpation if recognized to be macroscopically involved by transperitoneal palpation, positive by inspection if recognized to be macroscopically involved by palpation after opening retroperitoneal area, and positive by dissection if recognized to be macroscopically involved any time after starting the actual process of lymph node dissection
Eltabbakh	One American board-certified gynecologist oncolgist	-Lymphadenectomy -Lymph node sampling -Lymph node biopsy	-	-	-Lymph nodes were evaluated after opening the retroperitoneal spaces in the pelvis and the peritoneum over the lower vena cava and aorta and identifying the major blood vessels -In all cases, three signs of possible lymph node involvement by metastatic disease were evaluated: enlargement, firmness and adherence to surrounding structures -In addition, the surgeon recorded an overall impression on the basis of these three signs as to whether the lymph nodes were involved
Harter (2007)	3 Experienced gynecologist oncologists	Systematic pelvic and para-aortic lymphadenectomy	-Early ovarian cancer + primary complete cytoreductive surgery -Advanced ovarian cancer + primary complete cytoreductive surgery (residual disease smaller than 1 cm in 2000–2004 and macroscopic complete debulking after 2004)	-Resection of lymph nodes in the following regions: upper para-aortic region, lower para-aortic region, interaorto-caval region, para-caval region, iliaca communis, externa and interna regions and fossa obturatoria region	Intraoperative palpation
Khunnarong	Experienced gynecologist oncologists	-Lymphadenectomy -Lymph node sampling -Lymph node biopsy	-	-Pelvic lymphadenectomy: the deep circumflex iliac veins distally, the obturator nerves inferiorly, the internal iliac arteries medially and the aortic bifurcation proximally -Para-aortic lymphadenectomy: bifurcation of the aorta distally and the inferior mesenteric artery proximally	-Lymph nodes were evaluated after opening the retroperitoneal spaces in the pelvis and the peritoneum over the lower vena cava and aorta and identifying the major blood vessels -In all cases, the characteristics of lymph node metastasis that were evaluated included: size, consistency, shape and adherence to surrounding structures. These four characteristics were judged by individual surgeon. -In addition, the overall impression on the basis of these four characteristics as to whether the lymph nodes were involved were recorded

- Data not available.
